# A method for treatment monitoring using circulating tumour DNA in cancer patients without targetable mutations

**DOI:** 10.18632/oncotarget.25779

**Published:** 2018-07-24

**Authors:** Christina Demuth, Anne Winther-Larsen, Anne Tranberg Madsen, Peter Meldgaard, Boe Sandahl Sorensen

**Affiliations:** ^1^ Department of Clinical Biochemistry, Aarhus University Hospital, Aarhus, Denmark; ^2^ Department of Oncology, Aarhus University Hospital, Aarhus, Denmark

**Keywords:** circulating tumour DNA, non-small cell lung cancer, next-generation sequencing, digital PCR, wild-type EGFR

## Abstract

**Background:**

The potentials of circulating tumour DNA (ctDNA) have been studied for non-invasive disease monitoring in patients with targetable mutations. However, the majority of cancer patients harbour no targetable mutations. A workflow including targeted next-generation sequencing (NGS) and droplet digital PCR (ddPCR) could be used for monitoring treatment in these patients. Thus, our aim was to evaluate the workflow for ctDNA monitoring in a cohort of non-small cell lung cancer patients.

**Methods:**

Forty patients were prospectively included. Plasma samples were collected prior to and during treatment. NGS (Ion AmpliSeq Colon and Lung Cancer panel v2) was performed on ctDNA from pre-treatment samples. The identified mutations were monitored by ddPCR in consecutively collected samples.

**Results:**

Mutations were detected in 21 patients. The most commonly mutated genes were *TP53* (*N*=20) and *KRAS* (*N*=13). Treatment was discontinued due to non-response in 18 patients. In 16 of these, a simultaneous increase in ctDNA concentration was observed. A twofold ctDNA concentration increase confirmed in a second successive sample predicted non-response on the following imaging in 83% of patients (10/12).

**Conclusion:**

ctDNA monitoring can be used for early detection of non-response in patients without targetable mutations, and therefore could supplement imaging data for treatment monitoring in this subset of patients.

## INTRODUCTION

The potentials of circulating tumour DNA (ctDNA) for non-invasive disease monitoring have been intensively studied during the last decade. The primary investigated ctDNA source is plasma, and the small double-stranded DNA fragments are shed both actively and passively from tumour cells to the blood stream [1–3]. Tumour-specific alterations can be measured in ctDNA and reveal important information on the genetic constitution of the tumour [4]. Thus, mutation-specific ctDNA analyses have been investigated for, among others, earlier detection of cancer disease, relapse after surgery and detection of treatment resistance mechanisms [5–8]. Further, the concentration of ctDNA has been demonstrated to correlate with the tumour burden [9–12], and studies have shown promising data on the use of quantitative ctDNA analysis for monitoring tumour dynamics during treatment of patients with various solid cancers [13–15]. Thereby, monitoring ctDNA may identify tumour changes earlier than with radiological visualization.

Recent advances in next-generation sequencing (NGS) and droplet digital PCR (ddPCR) have facilitated the detection as well as the quantification of ctDNA. NGS enables identification of multiple genetic alterations in each patient and is optimal for revealing the genetic constitution of the tumour. In contrast, ddPCR requires *a priori* knowledge on the genetic constitution but often has lower detection limits and markedly shorter workflows than NGS making this approach useful for monitoring. The combination of these methods could present a workflow incorporable in the daily clinical routine that meet the needs associated with monitoring treatment in patients with unknown genetic constitution of their tumour.

In non-small cell lung cancer (NSCLC) patients, ctDNA monitoring has primarily been investigated in the patient subset with drug-targetable mutations present in tumour tissue. Thus, studies have focused on detecting and monitoring alterations in epidermal growth factor receptor (*EGFR*) and rearrangements of the anaplastic lymphoma kinase (*ALK*) during treatment with targeted therapies [7, 15–17]. By monitoring the abundance of *EGFR* mutations using ctDNA, studies have found correlation between decreasing ctDNA levels and tumour response on conventional imaging [18–20]. Further, whether or not *EGFR* mutations in ctDNA are cleared to an undetectable level may be predictive of response to EGFR-TKI therapy [21–23]. Yet, the majority of NSCLC patients are *EGFR* and *ALK* wild type. These patients have no targetable mutations and no information on the genetic constitution of their tumours exists. In combination, these facts render monitoring of ctDNA less straightforward in this patient subset and could explain why information on the clinical utility of ctDNA analysis in these patients lacks. However, given the poor prognosis and lack of efficient second- and third-line treatment of this large patient subgroup, it is highly warranted to find a method that can lead to earlier detection of non-response and rapid discontinuation of ineffective treatment.

Therefore, the aim of this prospective study was to investigate whether ctDNA monitoring using a clinically relevant workflow including a targeted NGS panel and ddPCR could be used for treatment monitoring in a cohort of NSCLC patients with unknown genetic constitution of their tumour.

## RESULTS

### Patients

A total of 40 patients were included. Patient characteristics are shown in Table 1. The majority of patients had stage IV disease, a good performance status and had progressed on first-line chemotherapy. No patients were lost to follow-up. After a median follow-up time of 7.4 months (range 1.0-35.1), one patient was still alive. The median OS in all patients was 7.0 months (95% confidence interval (CI): 3.7-9.3).

**Table 1:** Patient and tumour characteristics (N=40)

Characteristics	
**Age, Median years (range)**	67 (48-81)
	**N (%)**
**Gender**	
Female	15 (38)
Male	25 (62)
**Performance status, ECOG**	
0	4 (10)
1	31 (78)
2	5 (12)
**Smoking status**	
Never	1 (2)
Former^a^	28 (70)
Current	11 (28)
**Stage**	
III	3 (7)
IV	37 (93)
**Histology**	
Adenocarcinoma	33 (83)
Squamous cell carcinoma	7 (17)
**EGFR mutations (only adenocarcinoma patients)**	
EGFR Wild-type	33 (100)
**ALK rearrangements (only adenocarcinoma patients)**	
ALK wild-type	22 (67)
Not tested	11 (33)
**Erlotinib treatment**	
1st line	1 (2)
2nd line	30 (75)
3rd line	9 (23)
**Prior treatment**	
1st line	
Carboplatin/vinorelbine^b^	22 (56)
Carboplatin/vinorelbine/bevacizumab^c^	17 (44)
2nd line	
Pemetrexed	5 (56)
Docetaxel	4 (44)

Abbreviations: ECOG, Eastern Cooperative Oncology Group, EGFR, epidermal growth factor receptor; ALK, anaplastic lymphoma kinase.

^a^: Former smoker was defined as having stopped smoking at time of diagnosis.

^b^: Carboplatin day 1 (AUC 5) and vinorelbine day 1 and day 8 (60-80 mg/m2 (p.o.)) every 3 weeks for a maximum of four cycles.

^c^: Bevacizumab (7.5 mg/m2 i.v. day 1) was given in combination with chemotherapy. Patients with disease control received subsequent maintenance therapy every 3 weeks until progression or toxicity.

### ctDNA analyses

Sequencing of the baseline sample succeeded in 36/40 patients (90%). At least one alteration was detected in 21 of the 36 patients (58%). In total, 41 alterations were identified with a median allele frequency of 2.5% (range: 1.0–71.1) (Table 2). The most frequently mutated genes were *TP53* (*N*=20) and *KRAS* (*N*=13). The median number of alterations identified in each patient was 2 (range 1-5). In the baseline sample, 93% (38/41) of the mutations were verified by ddPCR. We directly compared the allele frequencies obtained from sequencing and ddPCR and found median ratio of 0.95 (range: 0.80;1.11) of NGS compared to ddPCR, suggesting good quantitative agreement between the methods (see Supplementary Figure 1). The three non-verified alterations were two *KRAS* mutations (p.K16R and p.G12S, PT ID 54) and an *ERBB4* mutation (p.W171L, PT ID 34). Though, the *ERBB4* mutation reappeared later in the treatment course (see Supplementary Figure 2). From the 21 patients with detectable molecular alterations at baseline, a total of 80 plasma samples were available with a median of 4 samples (range 2-12) from each patient. All ddPCR data is available in Supplementary Table 1.

**Table 2:** NGS and ddPCR results

Patient ID	Ion torrent PGM (Colon and lung panel)					Droplet Digital PCR	
	Gene	Mutation		Allele coverage (Ref coverage)	AF (%)	Fractional abundance (%)	Target molecules (copies/mL plasma)
		CDS	Protein				
4	MET	c.3328G>A	p.V1110I	16 (1667)	1	1.6	52.8
	TP53	c.833C>G	p.P278R	27 (1536)	1.8	2.6	96.8
6	KRAS	c.34G>A	p.G12S	40 (3201)	1.2	0.9	26.4
9	KRAS	c.34G>A	p.G12S	86 (3387)	2.5	1	26.4
	TP53	c.714_715insT	p.N239fs^*^1	339 (5734)	5.9	8.9	176
10	TP53	c.313G>T	p.G105C	32 (2800)	1.1	4.2	268.4
12	KRAS	c.34G>T	p.G12C	66 (3084)	2.1	2.2	264
13	KRAS	c.34G>A	p.G12S	7114 (9999)	71.1	72.8	74800
14	KRAS	c.34G>A	p.G12S	153 (3003)	5.1	2.9	226.6
	TP53	c.313G>T	p.G105C	343 (2750)	12.5	11.4	497.2
	SMAD4	c.1051G>A	p.D351N	612 (4554)	13.4	15.1	836
15	TP53	c.799C>T	p.R267W	82 (3961)	2.1	3.2	228.8
	STK11	c.766G>T	p.E256^*^	168 (6164)	2.7	2.1	123.2
21	KRAS	c.35G>A	p.G12D	426 (6666)	6.4	10.1	682
	KRAS	c.34G>A	p.G12S	1913 (8580)	22.3	24.7	1980
	TP53	c.491A>C	p.K164T	95 (5420)	1.8	4	162.8
	TP53	c.478A>G	p.M160V	105 (5363)	2	3.1	149.6
26	MET	c.3029C>T	p.T1010I	1994 (5643)	35.3	34.1	1738
	TP53	c.578A>G	p.H193R	662 (2674)	24.8	26.7	1452
29	EGFR	c.2235_2249del15	p.E746_A750delELREA	140 (4149)	3.4	2.1	129.8
	EGFR	c.2240T>C	p.L747S	60 (4178)	1.4	0.7	39.6
	TP53	c.641A>G	p.H214R	1214 (4574)	26.5	34.1	2706
30	TP53	c.730G>T	p.G244C	393 (5693)	6.9	9	154
34	ERBB4	c.512G>T	p.W171L	61 (2605)	2.3	0	0
	KRAS	c.34G>T	p.G12C	51 (1517)	3.4	5.5	52.8
	TP53	c.716A>G	p.N239S	112 (4539)	2.5	3.8	35.2
35	TP53	c.711G>A	p.M237I	60 (4353)	1.4	0.8	46.2
36	PIK3CA	c.1624G>C	p.E542Q	2250 (9973)	22.6	21.6	17952
	TP53	c.404G>A	p.C135Y	1716 (2745)	62.5	61.3	13684
38	KRAS	c.34G>T	p.G12C	40 (3081)	1.3	2	206.8
40	KRAS	c.34G>A	p.G12S	71 (3743)	1.9	3.2	149.6
	TP53	c.830G>T	p.C277F	37 (3210)	1.2	0.6	39.6
	TP53	c.742C>T	p.R248W	256 (7006)	3.7	4	257.4
	TP53	c.734G>A	p.G245D	70 (7021)	1	1.2	81.4
	TP53	c.578A>G	p.H193R	60 (4570)	1.3	0.8	52.8
51	KRAS	c.34G>T	p.G12C	46 (4237)	1.1	2.4	105.6
54	KRAS	c.47A>G	p.K16R	47 (3208)	1.5	0	0
	KRAS	c.34G>A	p.G12S	86 (3214)	2.7	0	0
	TP53	c.488A>G	p.Y163C	50 (2946)	1.7	1.2	259.6
57	TP53	c.715A>G	p.N239D	66 (6856)	1	1.4	33
64	TP53	c.844C>G	p.R282G	53 (1814)	2.9	1.3	336.6

Abbreviations: ^*^, translation termination; AF, allele frequency; CDS, coding DNA sequence; ddPCR, droplet digital PCR; del, deletion; fs*1, frame shift by insertion of 1 nucleotide; ins, insertion; NGS, Next generation sequencing; PGM, personal genome machine; ref, reference.

The majority of the sequencing data has been previously published [9].

### ctDNA concentration changes from baseline to radiological evaluation

**Figure 1:**
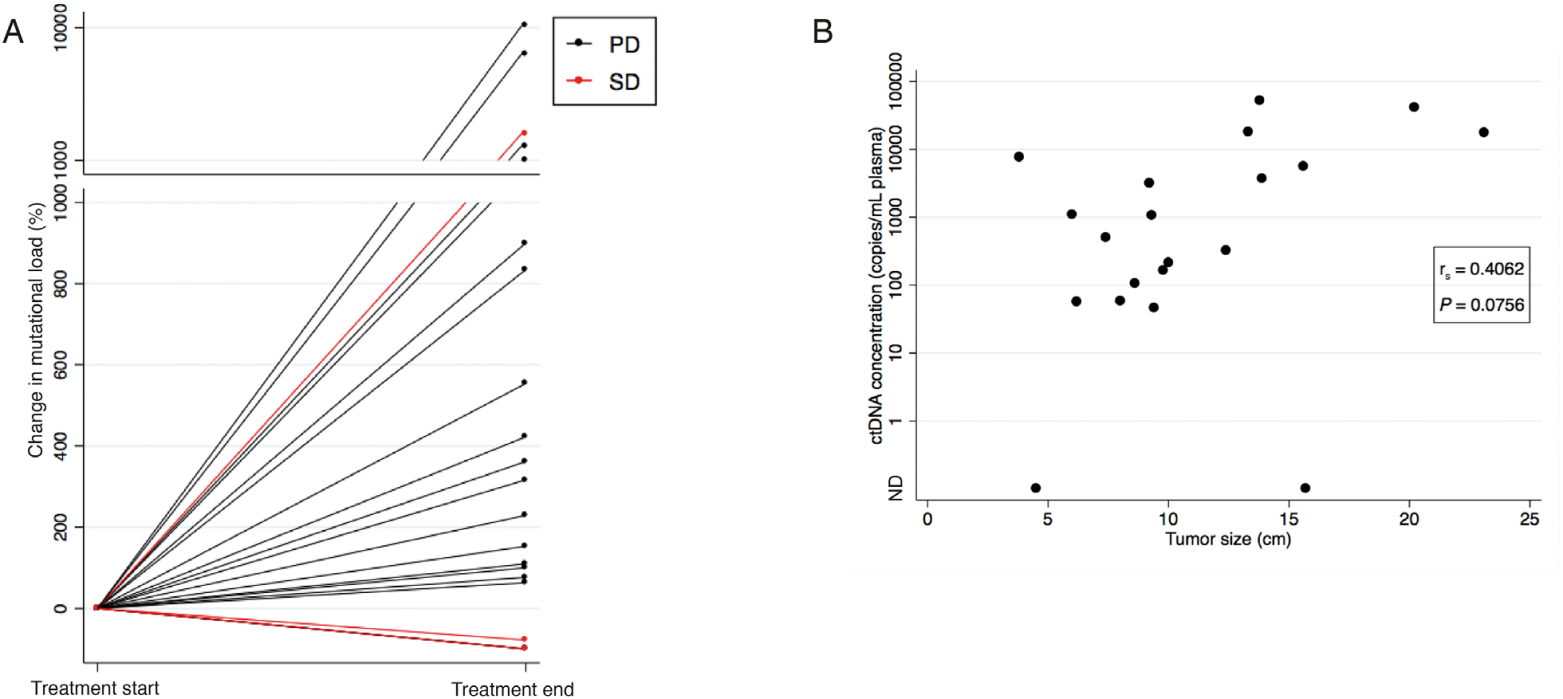
Correlation between ctDNA concentration and evaluation scan **(A)** The percentage change in ctDNA concentration from the baseline sample to the sample drawn at time of the last radiological evaluation illustrated for all 21 patients. Patients with radiological progression on the last evaluation scan (CT or MRI) are illustrated with black lines and patients with radiological stable disease (CT) are illustrated with red lines. Note the break in the y-axis, and the change in intervals. **(B)** Scatter plot showing the correlation between tumour size determined on the evaluation CT scan and ctDNA concentration at time of the CT evaluation in the 20 patients with an available CT evaluation scan. If various mutations were identified in a sample, the mutation with the highest concentration at progression was used. The *P*-value was calculated using Spearman’s correlation coefficient. Abbreviations: CT, computed tomography; MRI, Magnetic resonance imaging; ND, Not detectable; PD, Progressive Disease; SD, Stable Disease.

For 18 of the 21 patients, treatment was discontinued due to non-response detected on either a CT scan or a MRI of cerebrum. The percentage change in ctDNA concentration from the baseline sample to the sample drawn at time of the last radiological evaluation was evaluated. As can be seen from Figure 1A, 16 of these 18 non-responders presented with a simultaneously concentration increase for at least one mutation.

### ctDNA concentration monitoring

**Figure 2:**
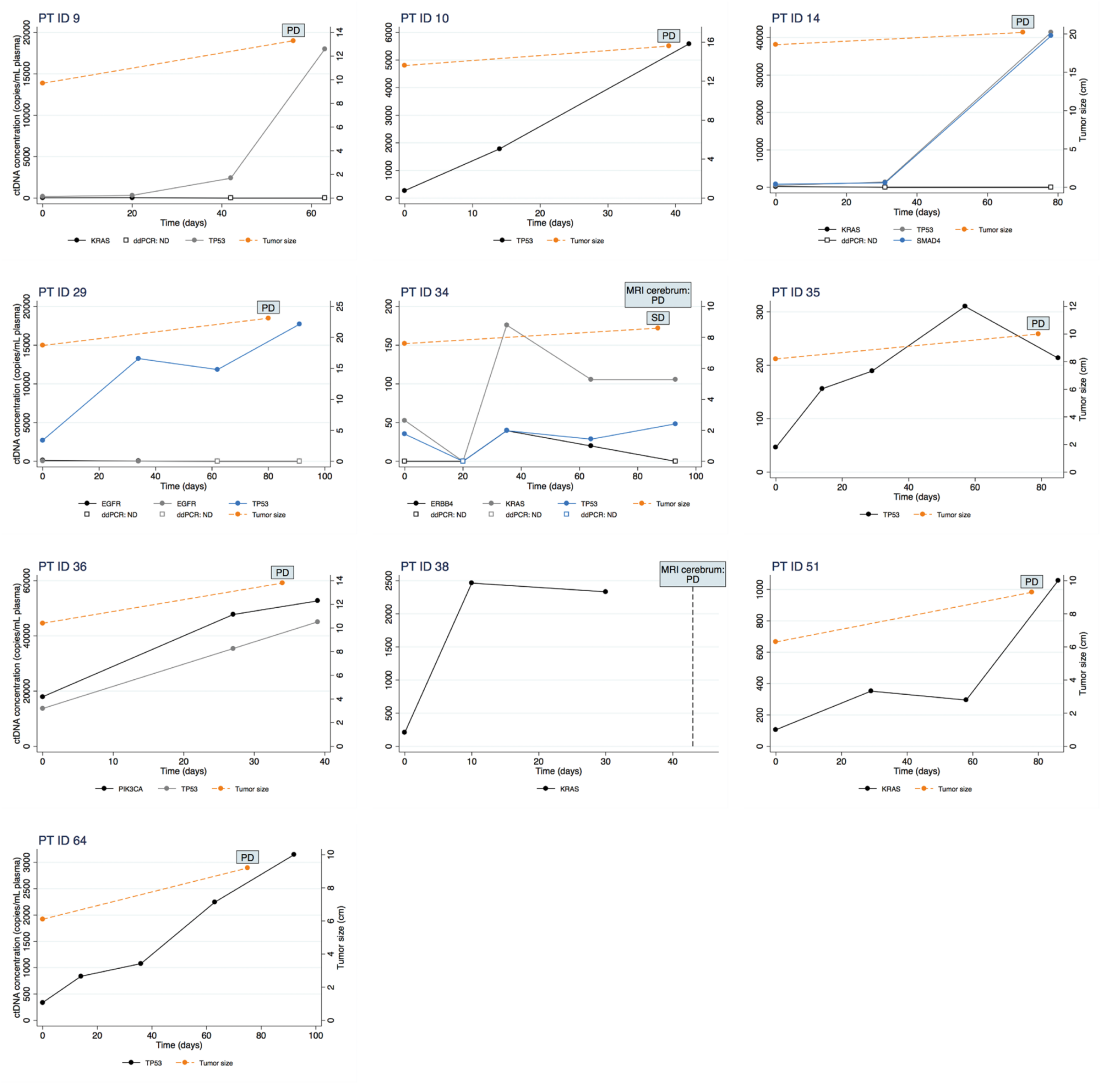
Changes in ctDNA concentration measured by ddPCR (left y-axis) during erlotinib treatment is illustrated for ten patients Time is depicted on the x-axis as days since start of treatment. Further, change in tumour size measured on a CT scan (right y-axis) is illustrated in the nine patients in whom a CT scan was performed. Abbreviations: CT, computed tomography; MRI, Magnetic resonance imaging; PD, Progressive Disease; SD, Stable Disease.

In order to investigate if non-response could have been predicted using ctDNA analysis, an increase in ctDNA concentration was defined as a twofold or higher percentage increase compared to baseline. Further, the twofold increase should be confirmed in a second successive sample. Sixteen patients had blood samples drawn during treatment in addition to those drawn at baseline and progression, and in these patients, we observed a maintained twofold increase in ctDNA concentration in 12 patients. For 10 of these (83%), radiological progression was detected on the following imaging. Among these 10 patients, eight patients already had a twofold increase in ctDNA concentration in the first blood sample collected after only a median of 21 days (range 14-34) of treatment. The results of the ctDNA monitoring along with changes in tumour size measured on CT scans are illustrated for each of these ten individual patients in Figure 2 and for the remaining in Supplementary Figure 2.

### ctDNA concentration in correlation to tumour size

For 20 out of 21 patients, at least one CT evaluation scan was available and tumour size was calculated on the last scan performed during erlotinib treatment. A median size of 9.6 cm (range 3.8-23.1) was found. The tumour size was correlated to the ctDNA concentration found in the sample drawn at the time of the CT evaluation. If various mutations were identified in a patient, the mutation with the highest concentration at progression was used. The correlation approached but did not meet statistically significant (Spearman’s correlation r=0.4062, *P*=0.0756) (Figure 1B).

## DISCUSSION

In this prospective study, we investigated whether a clinically relevant workflow including a targeted NGS panel and ddPCR could be utilised for treatment monitoring in a cohort of NSCLC patients with unknown genetic constitution of their tumour. We used a targeted 22-gene NGS panel including the most frequent mutations known in NSCLC which has proven useful for mutation detection in plasma ctDNA in previous studies [14, 24, 25]. Detection of ctDNA was possible in 58% of the patients, which was a bit lower than the comparable studies. However, the comparable studies either used a refined analysis method [24], had different detection limits [25] or a mixed cancer cohort [14], which may explain the differences.

Several studies have been conducted in *ALK* [16] and, especially, in *EGFR* mutation-positive TKI-treated patients [7, 15, 17] suggesting ctDNA as an effective tool for monitoring clinical response and emergence of resistance. However, monitoring treatment response in these patients is more straightforward as changes in the concentration of the targeted mutation can be detected. In *EGFR* and *ALK* wild-type patients there are very often more than one mutation with known functional impact [26], and it is challenging to distinguish the biological significance of the identified mutations. Here, we demonstrate that the ctDNA analysis also provide important information when studying *EGFR* and *ALK* wild-type patients as we observed an increased ctDNA concentration from baseline to treatment discontinuation in 16 out of the 18 patients in whom discontinuation of treatment was due to radiological non-response. Interestingly, we found that a twofold ctDNA concentration increase confirmed in a second successive sample was an early indicator of progression in a substantial sub-set of the patients (10 out of 12 patients). In addition, an increase in the first available blood sample collected median 21 days after initiation of treatment predicted progression in 8 of these 12 patients. These findings suggest that a sustained increase in ctDNA could be a very important indicator of disease progression and could indicate that an expedited radiological evaluation should be performed.

Important issues to consider regarding the use of ctDNA analysis is the cost and time of the procedure and thereby the feasibility of transferring the method into the daily clinic. Results are required within a few days for optimal use of ctDNA analysis for treatment monitoring by clinicians. However, an important challenge with NGS has been the need for time-consuming bioinformatics. With the development of targeted NGS, where a panel of predefined clinically relevant genes is sequenced, the laboratory workflow has been markedly reduced, making incorporation into the daily clinical routine achievable. In combination with ddPCR, this set-up offers a relative inexpensive and fast approach with a workflow of only one to four days, which is doable in a daily clinical setting.

This cohort of wild-type *EGFR* patients was treated with erlotinib while the current recommendations advice chemotherapy or immunotherapy in first- and second-line. However, non-targetable mutations were used for the monitoring leaving the treatment extraneous, and our results are comparable with studies of patients receiving other treatment regimens [17, 27].

In a recent study by Cabel et al. [27], the presence of ctDNA was evaluated during treatment with an immune checkpoint blocker in 15 cancer patients including 10 NSCLC patients, whereof 2 had an *EGFR* mutation (L858R). Concentrations of ctDNA were measured at initiation of therapy and again after 8 weeks of treatment, and in line with us, they found a correlation between the ctDNA concentration change and tumour response in the 10 patients with detectable ctDNA. This indicates that our findings are applicable to other treatment regimens.

A correlation approaching but not meeting statistically significance between tumour size and ctDNA concentration was observed. This correlation has been observed in several other studies [10–12], and we expect that the relatively low number of patients in our study may have impacted the calculation. Further, here we used a CT-defined tumour size which may not be the most optimal measurement for the entire tumour burden [9].

This study is the first of its kind and strengthened by the prospective nature. We evaluated a homogeneous patient cohort receiving the same treatment and with complete clinical data. Yet, the study has some limitations to consider. The number of patients was limited and our results are primarily hypothesis generating. Moreover, the biological variation of ctDNA is not well studied and could potentially influence our results. A recent study found no significant alterations in *EGFR* mutations detected in ctDNA by ddPCR at three time-points within one day (*N*=22) [28]. However, the day-to-day variation has not yet been studied.

## MATERIALS AND METHODS

### Patients

Advanced-stage NSCLC patients were prospectively enrolled in a single-centre study at the Department of Oncology, Aarhus University Hospital, Denmark from April 2013 until August 2015. Details on the study design have been published previously [29]. In short, patients were eligible for enrolment if the following criteria were fulfilled: histologically or cytologically proven NSCLC, age ≥ 18 years, performance status ≤ 2 and no prior tyrosine kinase inhibitor (TKI) treatment. All patients provided informed, written consent before inclusion. The study was approved by the Central Denmark Region Committees on Biomedical Research Ethics (no. 1-10-72-19-12) and performed in accordance with the Declaration of Helsinki.

Patients were treated with erlotinib in a palliative setting, and treatment was continued until radiological or clinical progression, unacceptable toxicity or death. A baseline computed tomography (CT) scan of the chest and abdomen was performed on all patients before erlotinib start. An evaluation CT scan was performed after 9-11 weeks of treatment or earlier on clinical indication. During the treatment course, additional CT scans were performed every 12 weeks. Neuroimaging with magnetic resonance imaging (MRI) was performed on clinical indication. Radiological response was quantified as percentage change in sum of longest diameter of target lesions according to Response Evaluation Criteria in Solid Tumours (RECIST) version 1.1 criteria [30]. Tumour size was calculated by summing the diameter of up to five target lesions in each patient according to the RECIST 1.1. Blood samples were consecutively collected before treatment start (baseline sample) and monthly during treatment until progression of disease.

As part of the routine diagnostic work-up, *EGFR* mutation testing was performed in all patients with adenocarcinoma (Therascreen EGFR RGQ PCR kit, QIAGEN, Manchester, UK). During the inclusion period, *ALK* rearrangement testing was incorporated as part of the routine diagnostic work-up in adenocarcinoma patients and was performed using IHC (screening) and Fluorescence *in situ* hybridization (verification).

In this present study, patients were considered eligible for inclusion if the following criteria were met: EGFR wild-type tumour, at least two blood samples available, a response evaluation CT or MRI scan had been performed and no more than three weeks between the last blood sample and the radiological evaluation. Inclusion of patients is illustrated in Supplementary Figure 3.

Blood samples collected from anonymous blood donors were used for investigating limit of detection (LoD) of the ddPCR. These were collected from the blood bank at Aarhus University Hospital.

### DNA extraction from blood

Ten millilitres of blood were collected in an EDTA-containing tube at each blood sampling. Within 2 hours of withdrawal, samples were centrifuged (1400 g for 15 min). Plasma was isolated and subsequently frozen at -80°C until further analysis. Cell-free DNA (cfDNA) was extracted from a range of 1-2 mL of patient plasma and from 4-5 mL of donor plasma using the QIAamp Circulating Nucleic Acid kit (Qiagen, Hilden, Germany) according to the manufacturer’s protocol. Elution of cfDNA was performed in 100 μL elution buffer supplied with the kit. The amount of cfDNA was quantified by measuring the beta-2-microglobulin (*B2M*) gene by ddPCR as described in Supplementary File 1 [29].

### Sequencing

The Oncomine™ Solid Tumour DNA kit (OST, Thermo Fisher Scientific), a CE-IVD-marked version of the Ion Ampliseq Colon and Lung Cancer Research Panel v2 (referred to as the Colon and Lung panel), was used to prepare sequencing libraries from cfDNA (1.1-10 ng). Sample preparation was performed using the Ion Chef™ Instrument, and sequencing was conducted on the Ion Personal Genome Machine® (PGM™) System (both Thermo Fisher Scientific, Watham, MA; USA). Each Ion 316™ v2 BC chip was loaded with eight samples. If samples did not meet the criteria of mean depth ≥ 2000, they were disqualified. Variants were called if they were exonic, previously observed, reported to COSMIC, and if the allele frequency ≥ 1% [14]. Benign SNPs were not reported. All variants were visualized and manually inspected using the Integrative Genomics Viewer [31]. The data analysis is described in Supplementary File 1.

The performances of each of the 92 amplicons were evaluated by investigating the mean coverage over the 36 patient samples successfully sequenced. Five amplicons generally performed poorly and did not meet the defined mean coverage (CHP2_ERBB4_1, CHP2_PTEN_2, ON_DDR2_3, CHP2_AKT1_1, CHP2_NOTCH1_1). These were excluded from further analyses. The majority of the sequence data has been previously published [9].

### Droplet digital PCR

The ddPCR reactions were performed using the QX200™ AutoDG™ Droplet Digital™ PCR System (Bio-Rad, Hercules, CA, USA). Conditions for ddPCR reactions are described in Supplementary File 1. All assays were purchased from Bio-Rad, and all relevant information can be found in Supplementary Table 2. When available, wet-lab validated assays were used. For the remaining, assays were designed by Bio-Rad and optimized in our laboratory. Assays were designed to run with an annealing temperature of 55 °C. In non-optimal cases, assays were tested using a temperature gradient to improve performance. Further, assays were tested on dilutions of the positive controls in order to test linearity and to choose the optimal concentration of the positive control. LoD for each individual assay was determined as described by Milbury et al. [32].

### Statistical analysis

To investigate the correlation between measurements obtained from NGS and ddPCR, llinear regression was performed. Agreement between methods was investigated using Bland-Altman plots. Log-transformed data was back-transformed to achieve median ratios. Spearman’s correlation was used to evaluate the correlation between ctDNA concentration and tumour size. Overall survival (OS) was determined as the time from start of erlotinib treatment until death of any course or last follow-up date (14^th^ august 2017). The estimate of median OS was calculated using the Kaplan–Meier method and the median follow-up time by the inverse Kaplan–Meier method. All tests were two-sided, and *P*-values less than 0.05 were considered to be statistically significant. Statistical analyses and graphic artwork were performed using STATA version 13 (Stata Corporation).

## CONCLUSIONS

In this study, we demonstrated that quantitative ctDNA monitoring by targeted NGS and ddPCR could be used for early detection of non-response in patients with unknown genetic constitution of their tumour. Particularly, a twofold ctDNA concentration gain in two successive blood samples was found to be an early indicator of progression. If these data are validated, ctDNA analysis could in the future supplement imaging data acquired by CT scans for treatment monitoring in this patient subset. While the prognosis is generally poor for this patient subgroup, a non-invasive method that could improve identification of non-responding patients early after treatment initiation could lead to earlier discontinuation of ineffective treatment. This will markedly reduce the risk of unnecessary toxicity and increase the chance of receiving other potentially effective treatments before worsening of performance status.

## SUPPLEMENTARY MATERIALS FIGURES AND TABLES







## Abbreviations

ALK: anaplastic lymphoma kinase
B2M: beta-2-microglobulin
cfDNA: cell-free DNA
CT: computed tomography
ctDNA: circulating tumor DNA
ddPCR: droplet digital PCR
EGFR: epidermal growth factor receptor
LoD: limit of detection
MRI: magnetic resonance imaging
NGS: next-generation sequencing
NSCLC: non-small cell lung cancer
OS: overall survival
RECIST: Response Evaluation Criteria in Solid Tumors
TKI: tyrosine kinase inhibitor
