# Supplementary material for: A method for treatment monitoring using circulating tumour DNA in cancer patients without targetable mutations
Source: Oncotarget. 2018 Jul 24;9(57):31066–76. doi: 10.18632/oncotarget.25779 (PMC6089565; doi:10.18632/oncotarget.25779)
Supplement: Supplementary file 1 [file oncotarget-09-31066-s001.pdf]

A method for treatment monitoring using circulating tumour DNA in cancer patients without targetable mutations

SUPPLEMENTARY MATERIALS

SUPPLEMENTARY FILE 1

cfDNA quantification

The *beta-2-microglobulin* (*B2M*) gene was quantified by droplet digital PCR (ddPCR) in duplicates. Primers and probes for quantification was: 5'-TAA AAC TTA ATG TCT TCC TTT TTT TTC TC-3' (forward), 5'-AAA CAT TTT CTC AAG GTC AAA AAC TTA-3' (reverse) and HEX/ZEN- 5'-CCT CCA TGA TGC TGC TTA CAT GTC TC-3'-IBFQ (probe). ddPCR amplification was performed in a 20 µl multiplex reaction containing 5 µl of purified cfDNA, 900 nM of primers, 200 nM of probe and 2X ddPCR Supermix for probes (no UTP).

Cycling steps for the ddPCR was as follows: initially an enzyme activation at 95˚C for 10 min (1 cycle) followed by 40 cycles of denaturation and annealing (each cycle at 94˚C for 30 s and 60˚C for 1 min) and finally enzyme deactivation at 98˚C for 10 min (1 cycle).

Sequencing

Signal processing, base calling and read alignment was conducted using the Torrent Suite™ Software (version 5.0.4). The Ion AmpliSeq Colon and Lung Cancer panel v2 template (Thermo Fisher Scientific). Hg19 was used as reference. The AmpliSeq CHPv2 peripheral/CTC/CF DNA single sample workflow in the Ion Reporter™ Software (version 5.0) was used to call variants (Thermo Fisher Scientific). Reference and hotspot BED-files were supplied with the kit. Default settings were retained.

Droplet digital PCR conditions

Reactions were a mix of the 2X ddPCR Supermix for Probes (no UTP, Bio-Rad), the relevant assay and 5 µL cfDNA in a total reaction volume of 20 µL following the manufacturer's recommendations. Droplets were prepared using the QX200™ AutoDG™. PCR was performed on either the Mastercycler Nexus Gradient instrument (Eppendorf, Hamburg, Germany) or the GeneAmp PCR System 9700 instrument (Applied Biosystems, Darmstadt, Germany). Results were obtained from the QX200™ Droplet Reader, and analysis was performed as recommended by the manufacturer using QuantaSoft™ Software version 1.7.4. As positive controls, Gene Strands (Eurofins Genomics, Ebersberg, Germany) diluted in donor cfDNA were used.

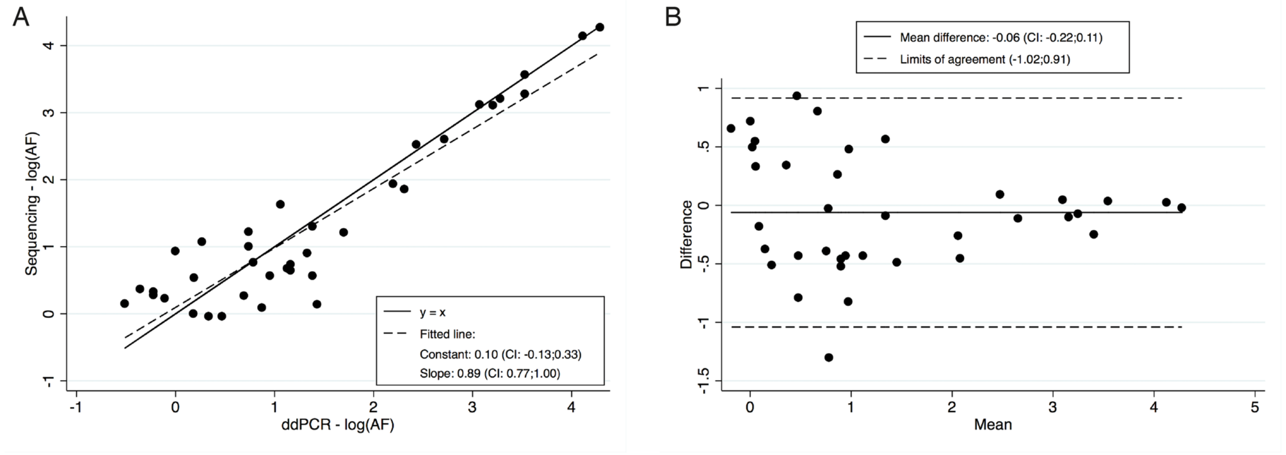

Supplementary Figure 1: Comparison of measurements obtained from NGS and ddPCR. **(A)** Linear regression was performed to investigate the correlation between measurements. **(B)** Bland-Altman plot was performed to investigate agreement between the methods. There is a tendency of lower agreement between the methods at low ctDNA AFs.

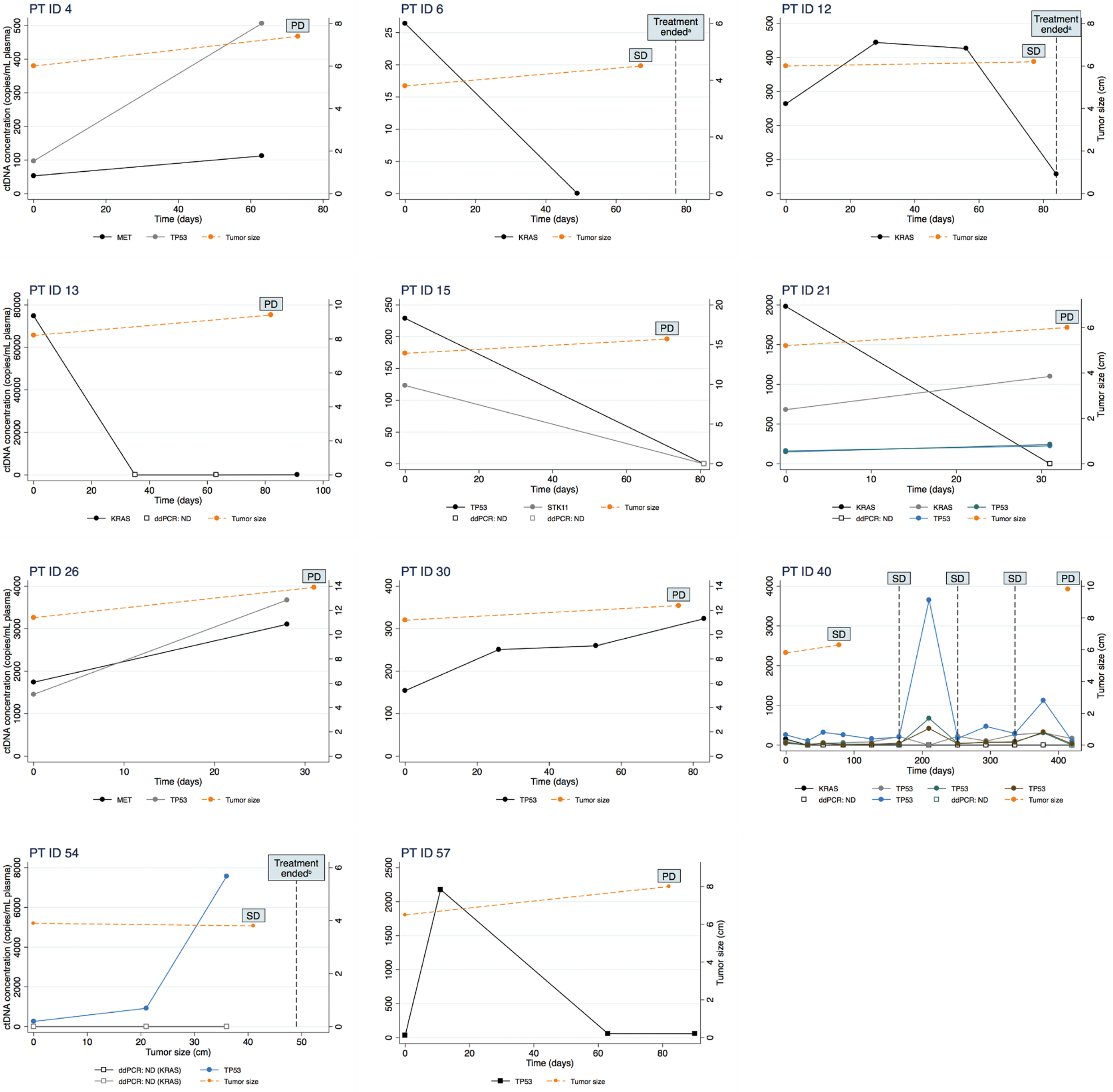

Supplementary Figure 2: Changes in ctDNA concentration measured by ddPCR (left y-axis) during erlotinib treatment are illustrated for 11 patients. Time is depicted as days after treatment start. Change in tumor size measured on a CT scan is illustrated (right y-axis).

A: Treatment ended due to worsening of performance status.

B: Treatment ended due to toxicity.

Abbreviations: PD, Progressive Disease; SD, Stable Disease.

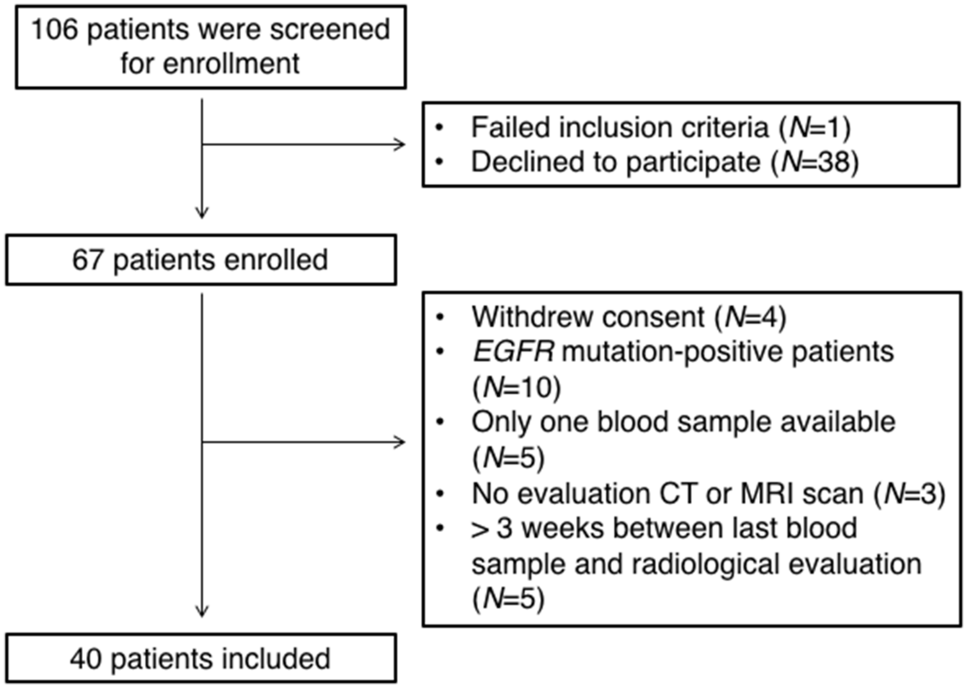

Supplementary Figure 3: Flow diagram of patient selection.

Supplementary Table 1: Complete ddPCR information on patient samples

See Supplementary File 1

Supplementary Table 2: ddPCR assay information

See Supplementary File 2
